# Audiomotor prediction errors drive speech adaptation even in the absence of overt movement

**DOI:** 10.1101/2024.08.13.607718

**Published:** 2024-08-16

**Authors:** Benjamin Parrell, Chris Naber, Olivia A. Kim, Caroline A. Nizolek, Samuel D. McDougle

**Affiliations:** 1 Waisman Center, University of Wisconsin–Madison; 2 Department of Communication Sciences and Disorders, University of Wisconsin–Madison; 3 Program in Neuroscience, Bates College; 4 Department of Psychology, Yale University; 5 Wu Tsai Institute, Yale University

## Abstract

Observed outcomes of our movements sometimes differ from our expectations. These sensory prediction errors recalibrate the brain’s internal models for motor control, reflected in alterations to subsequent movements that counteract these errors (motor adaptation). While leading theories suggest that all forms of motor adaptation are driven by learning from sensory prediction errors, dominant models of speech adaptation argue that adaptation results from integrating time-advanced copies of corrective feedback commands into feedforward motor programs. Here, we tested these competing theories of speech adaptation by inducing planned, but not executed, speech. Human speakers (male and female) were prompted to speak a word and, on a subset of trials, were rapidly cued to withhold the prompted speech. On standard trials, speakers were exposed to real-time playback of their own speech with an auditory perturbation of the first formant to induce single-trial speech adaptation. Speakers experienced a similar sensory error on movement cancelation trials, hearing a perturbation applied to a recording of their speech from a previous trial at the time they would have spoken. Speakers adapted to auditory prediction errors in both contexts, altering the spectral content of spoken vowels to counteract formant perturbations even when no actual movement coincided with the perturbed feedback. These results build upon recent findings in reaching, and suggest that prediction errors, rather than corrective motor commands, drive adaptation in speech.

## Introduction

Implicit motor adaptation is a prime example of the brain’s predictive capacities. In the standard framework, motor adaptation occurs when the predicted sensory consequences of a descending motor command are compared against feedback, and an internal model of those motor commands is adjusted to reduce observed discrepancies (i.e., sensory prediction errors) (Wolpert and Kawato, 1998; Wolpert and Flanagan, 2016; Krakauer et al., 2019). One lingering question in this framework is what serves as the input signal for the generation of sensory predictions that drive motor learning. In some models, that input signal is an efference copy generated from the descending motor command, which is then used to predict future states (Scott, 2004; Shadmehr and Krakauer, 2008; Houde and Nagarajan, 2011; Parrell et al., 2019). In other work, the input signal is defined as a higher-level motor plan (Day et al., 2016; Sheahan et al., 2016, 2018; McDougle et al., 2017; Vyas et al., 2020). It remains an open question whether motor planning or execution are sufficient for the computation of sensory predictions and errors, and whether there are common requirements for adaptation across motor subsystems.

In the case of speech adaptation, the prevailing idea is that ongoing feedback during actual execution of speech commands drives adaptation to audiomotor prediction errors (Tourville and Guenther, 2011; Guenther, 2016). Conversely, a recent model suggests that adaptation relies instead on auditory errors generated by the comparison of auditory reafference with auditory predictions generated by a task or planning-level controller rather than by motor efference (Kim et al., 2023a, 2023b). To date, it has been difficult to distinguish between these competing theories as they make similar predictions in existing speech adaptation paradigms. That said, several lines of evidence from upper-limb adaptation support the notion that an “upstream” motor plan is sufficient to drive adaptation. One piece of evidence for this is the discovery that internal signals preceding a movement, such as planning “lead-in” and “follow-through” motions, can partition otherwise catastrophically interfering motor memories (Sheahan et al., 2016, 2018). Indeed, a range of similar contextual pre-cueing effects have been observed in motor adaptation, supporting the idea that preparatory signals seed learning computations within the adaptation system (Wolpert et al., 2011; Howard et al., 2013; Heald et al., 2018, 2021; Vyas et al., 2020; Avraham et al., 2022; Churchland and Shenoy, 2024).

We have recently developed an experimental paradigm in limb control that is capable of teasing apart plan- vs. motor-based prediction error in sensorimotor adaptation, which we used to establish that implicit upper-limb motor adaptation could be induced in response to sensory errors that were unaccompanied by movements (Kim et al., 2022). Human subjects performed a “Go/NoGo” task that involved planning, but occasionally not executing, reaches to visual targets. On certain critical trials, subjects successfully inhibited reach commands but still observed a “virtual” sensory prediction error delivered via a visual cursor rotated relative to the planned reaching direction. These trials induced adaptation in subsequent movements, even though no movement had accompanied the error. In our view, these results offer particularly compelling support for the aforementioned plan-based model of adaptation.

Here we applied this design to speech adaptation in order to answer one of the major outstanding questions about sensorimotor adaptation in speech: whether ongoing feedback from executed speech movements is necessary for adaptation or whether speech adaptation can leverage sensory predictions based only on planned speech acts. Moreover, by expanding this paradigm from a laboratory reaching task to speech, an entirely novel motor domain that differs in several critical ways from limb control (bulbar vs. spinal control, hydrostatic control of the tongue vs. control of limb joint angles, auditory vs. visual feedback, etc), this work provides a critical test of the domain-generality of our previous findings. Similar results in speech and reaching would strongly support a shared neurocomputational mechanism driving sensorimotor adaptation.

## Results

**Figure 1:**
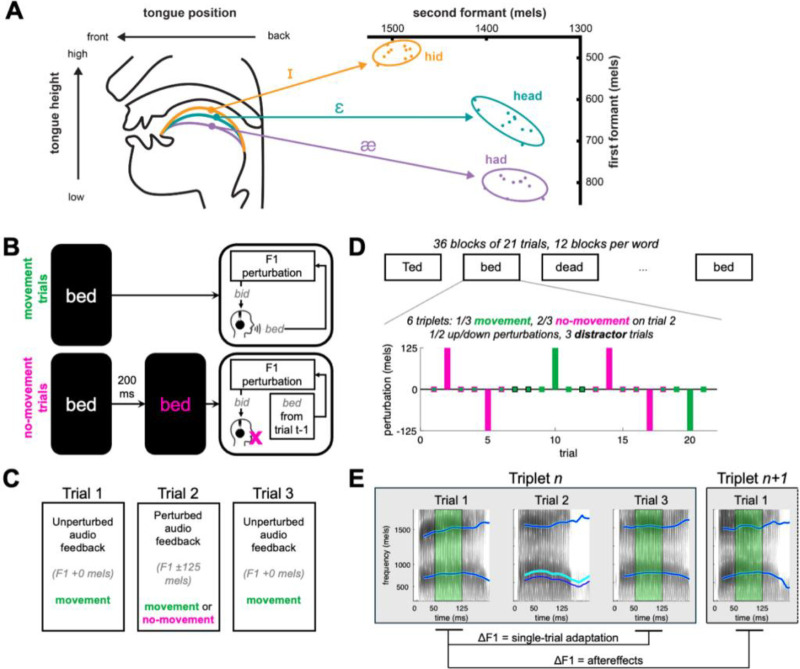
Experimental design. **A**: Illustration of the relationship between tongue position and vowel formants. The first two vowel formants (F1, F2) serve to distinguish between different vowels. **B**: Schematic of movement and no-movement trials. In movement trials, a stimulus word appeared on the screen and participants read the word out loud. Participants heard their own voice, with the first vowel formant (F1) perturbed or unperturbed. In no-movement trials, the stimulus word turned red 200 ms after appearing. Participants were instructed not to produce speech on these trials. Instead, the audio recording from the previous trial was played back to the participants with F1 perturbed. **C**: Triplet design. In each triplet, trials 1 and 3 were always movement trials with no perturbation applied. Trial 2 was variable – a movement or no-movement trial – and always had a perturbation applied. **D**: Experiment structure. The experiment consisted of 36 blocks which alternated between three stimulus words. Each block consisted of 6 triplets (2 with movement middle trials, 4 with no-movement middle trials) and 3 distractors (always movement trials) to disrupt the rhythm of the perturbations. **E**: Data analysis. Each trial shows a spectrogram of an example speech trial; cyan lines show produced vowel formants (F1 and F2) and blue lines show formants in headphone signal. For each trial, the produced F1 (the lowest frequency formant) was averaged between 50–125 ms after vowel onset (green shaded portion) to avoid coarticulatory effects of the initial consonant in each stimulus word and potential online compensatory effects. Single-trial adaptation was measured as the change in F1 from trial 1 to trial 3 within each triplet. Aftereffects were measured as the change from trial 1 of one triplet to the trial immediately following trial 3 of that triplet (either a distractor or trial 1 of the next triplet).

We implemented a task that allowed us to measure speech adaptation both with and without movement execution during perceived auditory prediction errors (Figure 1). Trials were organized into three-trial “triplets”, where the middle trial received a perturbation to the first vowel formant (F1), one of the resonances of the vocal tract determined by the position of the tongue, lips, and jaw which distinguish vowels from one another (Figure 1A). On “movement” trials, participants produced speech when cued by a visual stimulus. To measure adaptation in the absence of movement execution, we had participants occasionally inhibit a cued speech movement while still presenting them with a well-timed audiomotor prediction error (“no-movement” trials), using a formant perturbation applied to a recording of the speech produced on the immediately preceding trial. We measured adaptation for following both movement and no-movement trials as the change in F1 from the trial preceding the perturbed trial to the two trials immediately following the perturbed trial (adaptation and aftereffects, Figure 1E).

**Figure 2:**
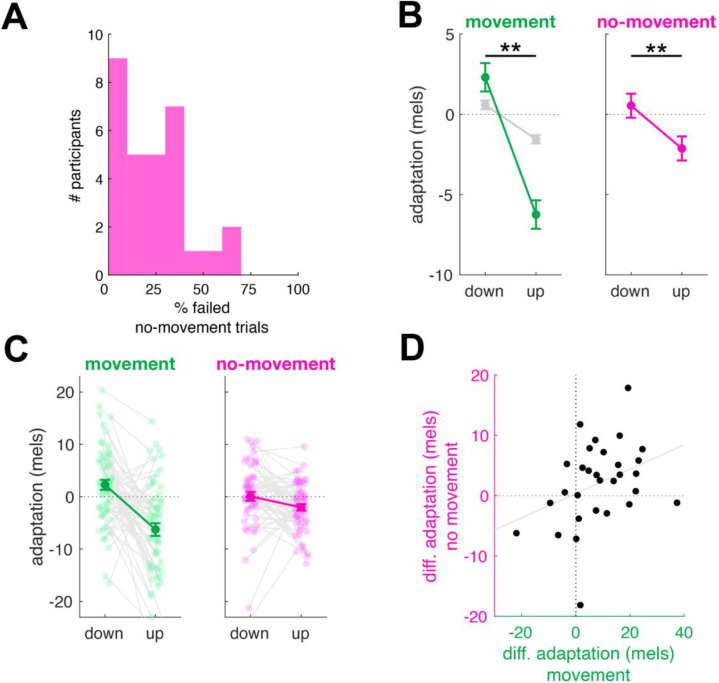
Experimental results for the adaptation index. **A**: The percentage of no-movement trials on which participants failed to inhibit vocal production. The average failure rate was 22% across participants. These trials were excluded from further analysis. **B**: F1 adaptation index (see Methods) following trials with downward and upward perturbations applied in movement (left) and no-movement (right) conditions. Estimated marginal means and standard errors shown in green and magenta. Gray indicates single-trial learning observed in Hatzsch et al. (2022). **C:** As for (B), showing data for individual participants. Means and standard error across participants are shown in dark, solid colors. Individual participant means shown as lighter colored dots connected by gray lines. **D**: Relationship across participants between the magnitude of differential adaptation (responses following downward perturbation minus responses following upward perturbations) in movement and non-movement conditions.

Despite the instruction to inhibit speech, participants nonetheless produced some acoustic speech material on a subset of no-movement trials (average of 22%, range 3–62%, Figure 2D). The prevalence of these failures suggests that our method was successful in driving participants to plan speech at the onset of the stimulus in these no-movement trials. In order to isolate learning in the absence of movement, only no-movement trials where participants successfully inhibited speech were used to measure adaptation.

Because the expected effect size of single-trial adaptation (~3 mels in Hantzsch et al., 2022, Fig 2A) is much smaller than the expected standard deviation of F1 in typical speech (~10–15 mels), our primary outcome measurement combined measurements taken from the third trial of each triplet immediately following the perturbation (adaptation) as well as the subsequent trial (aftereffects), in order to increase the power of our analysis and reduce noise. Participants produced robust single-trial learning following perturbations to both movement and no-movement trials (Figure 2A,B, main effect of perturbation direction F(1,10724) = 59.25, p < 1×10^−13^, partial R^2^ = 0.005). For movement trials, F1 following trials with a downward F1 perturbation showed a significantly higher F1 (2.3 ± 0.8 mels) than following trials with an upward F1 perturbation (−6.2 ± 0.8 mels, t(10722) = 7.5, p <.0001, d = 0.23). Single-trial adaptation on movement trials was 3–4 larger than in our previous work (Hantzsch et al., 2022), likely reflecting better control of stimuli in the current paradigm (see Discussion).

Crucially, following no-movement trials, F1 was significantly higher in trials following downward perturbations (0.5 ± 0.7 mels) compared to upward perturbations (−2.1 ± 0.8 mels, t(10736) = 2.9, p = 0.004, d = 0.07). This result supported our hypothesis that speech adaptation could proceed without actual movement execution. The magnitude of adaptation was smaller in no-movement trials compared to movement trials, reflected by a significant interaction between movement condition and perturbation direction (F(1,10725) = 16.3414, p < 0.0001, partial R^2^ = 0.002). Overall, the magnitude of adaptation in no-movement trials was roughly one-third of that found in movement trials, similar to our previous results in adaptation of upper limb movements (Kim et al., 2022). (Potential reasons for this replicated drop in effect size are addressed in the Discussion.)

We then looked at a between-subjects correlation between adaptation effect sizes on movement versus no-movement triplets. Indeed, there was a moderate, though statistically marginal, positive relationship between learning in movement and non-movement trials across participants in the expected direction (Figure 2C, r = 0.36, p = 0.053).

**Figure 3:**
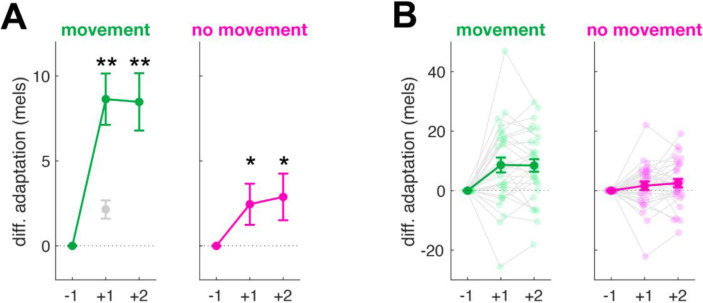
Experimental results separated by trial number. **A**: Differential adaptation shown separately for trials immediately following the perturbation (+1, adaptation) and the next trial (+2, aftereffects) in the movement (left) and no-movement (right) conditions. Estimated marginal means and standard errors shown in green and magenta. Gray indicates single-trial learning observed in Hatzsch et al. (2022). **B:** As for (A), showing data for individual participants. Means and standard error across participants are shown in dark, solid colors while individual participant means are shown as lighter colored dots connected by gray lines. * p < 0.05; ** p < 0.005.

As an additional analysis, we considered adaptation (in trials immediately following the perturbation, *t+1*) and aftereffects (in the next following trial, *t+2*) separately (Figure 3). The results for both adaptation and aftereffects were consistent with the combined results. For adaptation, there was a significant main effect of perturbation direction (F(1,5362.3) = 32.7, p < 1×10^−7^, partial R^2^ = 0.006) as well as a significant interaction between perturbation direction and movement condition (F(1,5362.3) = 10.2, p = 0.001, partial R^2^ = 0.002). The difference in F1 between upward and downward trials was significant for both movement (8.6±1.5 mels, t(5363) = 5.7, p < 0.0001, d = 0.24) and no-movement conditions (2.5±1.2 mels, t(5369) = 2.014 p = 0.04, d = 0.07), though the magnitude of this difference was again smaller for the no-movement condition.

The results for aftereffects were similar: Upward and downward perturbations led to differences in F1 in both movement (8.5±1.7 mels, t(5328) = 4.997 p < .0001, d = 0.22) and no-movement (2.9±1.4 mels, t(5341) = 2.110, p = 0.03, d = 0.07) conditions (main effect of perturbation direction: F(1,5355) = 27.2, p < 1×10^−6^, partial R^2^ = 0.005); and the magnitude of this difference was smaller in the no-movement condition (interaction: F(1,5355) = 6.6, p = 0.01, partial R^2^ = 0.001).

## Discussion

Here we demonstrated that speech adaptation can occur even when audiomotor errors are not accompanied by movement execution. First, we replicated the result that when participants were exposed to a perturbation of their first vowel formant on a single trial, changes in speech articulator movements that opposed that perturbation were visible on the two trials following the perturbation. These results confirm that single-trial speech adaptation observed in previous paradigms designed to examine online compensatory movements (Hantzsch et al., 2022) can be reliably elicited with our triplet design. Remarkably, adaptation occurred not only when participants heard an auditory perturbation during speech, but also when participants planned to produce speech, but withheld overt speech movement and instead heard playback of their own speech from a previous trial with the perturbation applied. That is, when participants putatively generated a movement plan and a concomitant auditory prediction, they adapted their movements to correct for observed audiomotor prediction errors even though they did not produce any overt speech movements in conjunction with those errors. These results extend, in a new motor and sensory domain, our recent findings in upper-limb visuomotor adaptation (Kim et al., 2022).

The current results suggest that audiomotor speech adaptation is driven, at least in part, by using sensory prediction errors to directly update internal models controlling speech articulators. Such updates are consistent with dominant models of sensorimotor learning in limb and oculomotor control (Wolpert et al., 1998; Smith et al., 2006; Shadmehr and Krakauer, 2008; Shadmehr et al., 2010; Hadjiosif et al., 2021). However, these results are inconsistent with the dominant model of sensorimotor adaptation in speech production, which suggests that adaptation is driven by the incorporation of corrective movements generated by a feedback controller in response to sensory errors into future feedforward motor programs (Tourville and Guenther, 2011; Guenther, 2016; Kearney et al., 2020), which itself is partially based on other ideas in limb control (Kawato et al., 1987; Albert and Shadmehr, 2016).

Notably, the magnitude of the change observed on the movement triplets (~8 mel difference between upward and downward perturbations) was roughly 2–3 times larger than we previously observed using the same analysis window (Hantzsch et al., 2022). Why did we see this large increase in effect size? One possibility is that our new design, which uses blocked repetition of the same stimuli, induces stronger learning signals than when stimuli are mixed across trials as in previous studies. This could be because adaptation in speech only partially generalizes to untrained words (Rochet-Capellan et al., 2012; Caudrelier et al., 2018), similar to the local spatial generalization of learning observed in reaching (Gandolfo et al., 1996; Krakauer et al., 2000; Donchin et al., 2003). Importantly, the larger effect size we observed in our movement trials may allow for more precise assays of factors that affect sensorimotor adaptation in speech, given that around 100 observations of learning can be obtained in the time it would typically take to obtain a single observation (e.g., mean asymptotic learning) using a more traditional paradigm with extended exposure to a repeated auditory perturbation.

Intriguingly, the size of the reduction we observed in speech adaptation between movement and no-movement trials (roughly one-third, Figures 2 and 3) was very similar to the reduction previously observed in a similar reaching task (Kim et al., 2022), pointing to a domain-general explanation. We see several possible explanations for this observed reduction: First, it has been established, both in speech and reaching tasks, that temporal delays between movement and sensory feedback substantially impair adaptation to errors (Kitazawa et al., 1995; Brudner et al., 2016; Schween and Hegele, 2017; Zhou et al., 2017). In speech, the magnitude of adaptation is reduced by roughly 50% when auditory feedback is delayed by only 100ms, and adaptation is essentially absent when delays reach 250–500ms (Max and Maffett, 2015; Shiller et al., 2020). This suggests that the critical comparison driving auditory error processing is highly temporally-specific, particularly in speech. In our paradigm it is impossible to know exactly when speech would have occurred on the no-movement trials; as an estimate, the latency of the speech feedback on a given no-movement trial was matched to the latency on a previous movement trial. This method is likely to have introduced variance between the timing of anticipated and perceived auditory feedback on no-movement trials, potentially leading to a reduction in the magnitude of adaptation.

Second, reductions in somatosensory feedback inherent in no-movement trials may have suppressed the degree of adaptation possible. It has been suggested that, in limb control, adaptation may result not from a drive to reduce visual sensory error but rather from the recalibration of somatosensory and other sensory signals (Tsay et al., 2022). Consistent with this idea, putatively disruptive noninvasive stimulation to primary somatosensory cortex following visuomotor reach adaptation substantially decreases how much of this learning is retained (Ebrahimi and Ostry, 2024). Thus, when errors occur in the absence of somatosensory feedback, such as in our no-movement trials, it is possible that adaptation magnitude would be reduced.

Finally, it may also be the case that adaptation in speech can be driven by auditory prediction errors both directly through updates to predictive internal models and indirectly through incorporation of previous feedback-based commands into feedforward motor programs. In fact, there is support for this “dual input” idea in tasks requiring adapting to dynamic force-field perturbations during reaching (Albert and Shadmehr, 2016). Thus, an important note here is that while many results point to a plan-based sensory prediction error model of adaptation, both planning and ongoing feedback commands could both provide inputs that generate sensory predictions. Future work can directly assay if and how both sources of information fuel adaptation.

An additional putative learning mechanism that may be connected to the current study is observational learning. It is possible that additional contributions from observation-based learning could be relevant here, as subjects in our task observed errors without moving (Mattar and Gribble, 2005; Pawlowsky et al., 2023). However, we believe it is unlikely that observational learning fully explains our results: In speech production, producing a word after an auditory presentation of the same word typically drives speakers to change their production to be *closer* to the auditory stimulus, a process known as “phonetic convergence” (Goldinger, 1998; Shockley et al., 2004; Mattar and Gribble, 2005; Babel, 2012; Babel and Bulatov, 2012; Pawlowsky et al., 2023).

Although direct neurophysiological data related to speech adaptation is limited, our results are consistent with invasive and non-invasive imaging studies that have examined sensory prediction in speech. When we speak, auditory cortical activity is suppressed relative to when we passively listen to the same sounds, a process thought to be driven by the cancellation of auditory reafference using predictive internal models (Curio et al., 2000; Houde et al., 2002; Ventura et al., 2009; Flinker et al., 2010). This suppression effect is reduced in less prototypical productions, suggesting that the prediction is based on a plan (or target) rather than motor efference (Niziolek et al., 2013; Tang et al., 2023; Beach et al., 2024). Recent work has shown that suppression is modulated during adaptation, and that the degree of modulation predicts the degree of learning, strongly suggesting that these predictions (and the resulting sensory prediction errors) play a critical role in adaptation (Kim et al., 2023b). Single-unit recordings in marmosets indicate that suppression of auditory cortex firing begins hundreds of milliseconds ahead of vocalization initiation (Eliades and Wang, 2003), and stimulus-specific cortical modulation has been demonstrated in this pre-vocalization window in humans (Daliri and Max, 2016), suggesting that vocalization planning alone can modulate auditory cortex activity and may be sufficient to support error computation.

While our results cannot directly answer whether such sensory errors drive learning through updates to forward models or control policies, they are nonetheless consistent with the general consensus that adaptation is driven by a cerebellar-dependent process based on sensory prediction errors (Bastian, 2006; Flanagan et al., 2003; Haith & Krakauer, 2013; Miall & Wolpert, 1996; Shadmehr et al., 2010; Wolpert & Kawato, 1998). Our results, along with our previous results in upper-limb adaptation, strongly suggest that these predictions are seeded, at least in part, by a higher-level planning process.

There are two caveats about our no-movement condition. First, although single-trial adaptation was robust in the no-movement condition, the overall effect was relatively small (~2 mels, d = 0.07). Though this magnitude is roughly equivalent to our previous work demonstrating single-trial adaptation (in movement trials) when stimulus words varied across trials (Hantzsch et al., 2022)), speech, in general, is substantially more variable than this, with a standard deviation in F1 of roughly 10–15 mels. Our triplet design allowed for a high number of observations per participant in order to look for this “needle in the haystack.” Second, while we excluded no-movement trials with any overt sound production of any kind, it is possible that participants nonetheless produced some subtle muscular activity on a subset of these trials. However, there is some evidence, though from a slightly different task, that articulatory movements and voicing frequently co-occur when stop signals occur at a similar latency after the go signal as in the current study (Tilsen and Goldstein, 2012). This suggests that the acoustic signal used as a criteria for exclusion here is likely to also exclude trials with overt movement – as such, we do not believe that potential latent muscular activity substantially changes the main conclusions we draw from these data. Nonetheless, future work could measure muscle activity or movement more directly, such as with surface EMG of the masseter or articulatory tracking of the tongue, to resolve this question more directly.

Overall, our study suggests that the direct updating of internal models through sensory prediction errors is sufficient to drive speech adaptation. In our view, speech planning may provide the critical sensory predictions that, when violated, lead to adaptation of internal models governing speech control. These results do not support models of speech adaptation that rely solely on the incorporation of feedback-based motor commands into future feedforward plans, suggesting that such models could be revised to incorporate direct updating of internal models through sensory error processing. Moreover, by extending our previous findings in upper-limb adaptation (Kim et al., 2022) to a novel motor domain (speech) and a different sensory system (audition), we show that plan-based predictions may form the basis for sensorimotor adaptation across a wide range of human motor behaviors, pointing to a shared, domain-general neurocomputational mechanism.

## Methods

### Participants and power

30 participants were tested in the current study (5 male/25 female, age range 18–45, mean age 23.4). The sample size was determined using a bootstrapping procedure with effect sizes observed in our previous work (Hantzsch et al., 2022), with the target of 90% power to detect a similar sized effect at α = 0.05. All participants were native speakers of American English, without any reported history of neurological, speech, or hearing disorders. All participants passed an automated Hughson-Westlake hearing screening (thresholds 25 dB HL or lower in both ears at 250, 500, 1000, 2000, and 4000 Hz). Participants gave written informed consent prior to participation in the study and were compensated either monetarily or with course credit. All procedures were approved by the Institutional Review Board of the University of Wisconsin–Madison (protocol 2017–1128).

### Task setup

Participants were seated in a sound-insulated booth in front of a computer monitor. On each trial, a target word appeared on the screen in white text (Figure 1B). Each trial lasted 1.7 s from stimulus onset. Trials were separated by 1.25 s plus a random delay of 0–0.5 s. Participants were instructed to read the words as quickly as possible as they appeared on each trial. Two trial types were used – “movement” trials and “no-movement” trials, following our previous study in reach adaptation (Kim et al., 2022). On the majority of trials (movement trials), the word stayed white for the duration of the trial. On a subset of trials (no-movement trials), the target word turned red 200 ms after it appeared and stayed red for the remainder of the trial. Participants were instructed to not produce overt speech if and when the target word turned red. 200 ms was chosen after pilot testing suggested that this delay results in the inhibition of speech on most, but not all, trials in the majority of participants. This delay was therefore long enough to elicit movement planning but short enough to enable mostly successful inhibition, thereby allowing us to test whether sensory feedback given in the absence of overt movement could drive speech adaptation.

On movement trials, participants’ speech was recorded (AKG C520), digitized with a USB sound card (Focusrite Scarlett 2i2), processed through the Audapter software package (Cai et al., 2008; Tourville et al., 2013), and played back to the participants over closed-back, over-the-ear headphones (Beyerdynamic DT 770). Speech was played back at a volume of approximately 83 dB SPL and mixed with speech-shaped noise at approximately 60 dB SPL. The final level of the playback speech signal varied with the amplitude of participants’ produced speech. The noise, combined with the closed-back headphones, served to minimize potential perception of the participants’ own unaltered speech, which may have otherwise been perceptible through air or bone conduction. The latency of audio playback on our system is ~18 ms, as measured using the protocol suggested by (Kim et al., 2020).

Trials were organized into “triplets.” The first and last trials of each triplet were always movement trials with veridical auditory feedback (Figure 1C). On the middle trial of each triplet, the auditory feedback participants received was always perturbed, such that the first vowel formant (F1, Figure 1A) was either raised or lowered by 125 mels (a perceptually-calibrated measurement of frequency) throughout the trial using Audapter. Middle trials were either movement trials (⅓ of triplets) or no-movement trials with a stop signal (⅔ of triplets). On perturbed movement trials, the auditory feedback was perturbed in real time by identifying the vowel formants using linear predictive coding (LPC) and filtering the speech signal to introduce a shift to those formants. On perturbed no-movement trials, Audapter was used offline to apply the same shift to a recording of the participants’ production from the previous trial (i.e. the first trial of the triplet, which was always an unperturbed “movement” trial). On these trials, Audapter was initiated at the same time as on movement trials, but played back this perturbed speech signal rather than playing back the speech recorded in real time. Thus, the latency of the auditory feedback on each no-movement trial was the same as on the immediately preceding trial.

Triplets were organized into blocks (Figure 1D). Each block contained 2 movement triplets and 4 no-movement triplets. Upward and downward frequency perturbations were balanced within each block. The order of triplets within each block was randomized. Each block additionally contained 3 distractor trials, randomly inserted between triplets, in order to disrupt the rhythm of the perturbations across trials. All distractor trials were movement trials with veridical auditory feedback. Each block used a single stimulus word (“bed”, “dead”, or “Ted”, all sharing the same target vowel /ɛ/); stimuli were pseudo-randomized across blocks such that there were an equal number of blocks for all stimuli, and no consecutive blocks used the same stimulus. The experiment consisted of 36 total blocks, yielding 216 total triplets. This resulted in 36 perturbations in movement blocks and 72 perturbations in no-movement blocks for each perturbation direction. A short self-timed break was allowed between each block.

In order to encourage participants to produce their speech movements quickly, they were given points based on their response latency on movement trials. 10 points were given for responses with latencies up to 500 ms, falling off by 1 point every 40 ms thereafter. To encourage participants to inhibit overt speech production on no-movement trials, −25 points were given when a spoken response was detected. On all trials, the onset of speech (and response latency) was determined as the point where the amplitude of the microphone signal crossed above a predetermined, low amplitude threshold.

### Data quantification

Formant data were tracked using wave_viewer (Niziolek and Houde, 2015), which provides a MATLAB GUI interface for formant tracking using Praat (Boersma and Weenink, 2019). LPC order and pre-emphasis values were set individually for each participant. Vowels were initially automatically identified by locating the samples which were above a participant-specific amplitude level. Subsequently, all trials were hand-checked for errors. Errors in formant tracking were corrected by adjusting the pre-emphasis value or LPC order. Errors in the location of vowel onset and offset were corrected by hand-marking these times using landmarks in the audio waveform and spectrogram. For each trial, F1 was averaged from 50–125 ms after vowel onset (Figure 1E) to avoid 1) coarticulatory influences on vowel formants from the initial consonant and 2) potential changes in vowel formants due to online feedback corrections, which begin roughly 150 ms after vowel onset (Cai et al., 2012; Parrell et al., 2017). Recent work has shown that this window is the most likely to accurately capture learning in this single-exposure paradigm (Hantzsch et al., 2022).

Adaptation was quantified in three ways. Single-trial adaptation was measured as the change in F1 (in mels) from the first to the third trial of a triplet (i.e., after exposure to the auditory perturbation). We additionally calculated single-trial aftereffects – the retention of learning – by measuring the change in F1 (in mels) from the first trial in a triplet to the trial immediately following the third trial of that triplet. Finally, we computed an “adaptation index,” which was quantified by simply combining the above two metrics for each triplet. In all cases, the critical trials were unperturbed movement trials, either the first trial of the following triplet or a distractor trial. Because of the rather high variance in F1 production for vowels (standard deviation of ~15–20 Hz, Whalen and Chen, 2019) relative to the maximum expected effect size given previous work (~2 Hz, Hantzsch et al., 2022), the adaptation index was our primary dependent variable of interest, designed to maximize our statistical power and potential to detect a small effect relative to the expected variance in production.

A small number of trials were excluded due to errors in production (i.e., the participant said the wrong word), disfluencies, or unresolvable errors in formant tracking (0–3.6% across participants, median .03%). Additionally, no-movement triplets where participants produced any detectable vocalization on the middle perturbed trial were excluded in order to isolate learning effects when no speech movement was produced (4–89 triplets excluded across participants, median 32). In total, these exclusions resulted in 64–72 independent measurements of adaptation (median 71) and 52–140 independent measurements of retention for each participant (median 111).

### Statistical analysis

Following our previous study in reaching (Kim et al., 2022), linear mixed effects models were run using the lme4 package in R (Bates et al., 2015). For single-trial adaptation, retention, and the adaptation index models, model predictors included perturbation direction (upward or downward), triplet type (movement or no-movement), and the interaction between these two factors. Participant was included as a random effect (intercept). Statistical significance was assessed using the lmerTest package (Kuznetsova et al., 2017). Post-hoc comparisons were conducted with estimated marginal means using the Satterthwaite method for approximating the degrees of freedom (Lenth et al., 2023). Effect sizes are reported as partial R^2^ values from the linear mixed effects models and Cohen’s *d* for pairwise comparisons. Summary statistics report means and standard errors.
